# Is There a Role of Beetroot Consumption on the Recovery of Oxidative Status and Muscle Damage in Ultra-Endurance Runners?

**DOI:** 10.3390/nu16050583

**Published:** 2024-02-21

**Authors:** Eva Vilar, Eladio Collado-Boira, Carlos Guerrero, Ana Folch-Ayora, Pablo Salas-Medina, Carlos Hernando, Pablo Baliño, María Muriach

**Affiliations:** 1 Hospital de La Plana, Vila-Real, 12540 Castellon, Spain; eva.vilar.yebra@gmail.com; 2 Unidad Predepartamental de Enfermeria, Jaume I University, 12071 Castellon, Spain; colladoe@uji.es (E.C.-B.); afolch@uji.es (A.F.-A.); psalas@uji.es (P.S.-M.); 3 Unitat Predepartamental de Medicina, Jaume I University, 12071 Castellón, Spain; cguerrer@uji.es; 4 Department of Education and Specific Didactics, Sport Service, Jaume I University, 12071 Castellon, Spain; hernando@uji.es

**Keywords:** oxidative stress, ultra-endurance sport, antioxidants, muscle damage

## Abstract

(1) Background: Ultra-endurance exercise involves a high physical impact, resulting in muscle damage, inflammatory response and production of free radicals that alter the body’s oxidative state. Supplementation with antioxidants, such as beetroot, may improve recovery in ultra-endurance runners. The aim of this study was to determine whether there is a correlation between beetroot intake and recovery of serum oxidative status, inflammatory response and muscle damage parameters after an ultra-endurance race. (2) Methods: An observational and longitudinal study was conducted by means of surveys and blood samples collected from 32 runners during the IX Penyagolosa Trails CSP^®®^ race and the two following days. The variables C-reactive protein (CRP), lactate dehydrogenase (LDH), creatine kinase (CK), the activity of the antioxidant enzymes glutathione peroxidase (GPx) and glutathione reductase (GR) as well as the oxidative damage markers malondialdehyde (MDA), carbonyl groups (CG) and loss of muscle strength using the squat jump (SJ) test were analyzed to discriminate whether beetroot consumption can modulate the recovery of ultra-trail runners. (3) Results: Significant differences were observed between runners who ingested beetroot and those who did not, in terms of oxidative status, specifically in serum GPx activity at 24 and 48 h, muscle damage variables CK and LDH and regarding the SJ test results at the finish line. Therefore, the intake of supplements containing beetroot positively influences the recovery of serum oxidative status and muscle damage after ultra-endurance running.

## 1. Introduction

Mountain running and ultrarunning are sports practices that have been the subject of study and analysis in Spain in recent years. While moderate physical activity has beneficial effects on health, the vigorous ultra-endurance exercise involved in these extremely long races constitutes a new challenge to be studied due to the acute consequences they have on the physiology of the human body [1,2]. It is well documented that among the effects of ultramarathon running are cellular damage at the muscular level, as well as alteration of the inflammatory response and antioxidant defenses [1,3,4], which are the objects of our study. If ultramarathon races are intense due to the distance involved, in the case of mountain races with large accumulated gradients, two characteristics are added that also influence oxidative stress and muscle damage: the effect of exercise at altitude [5] and the myofibrillar damage caused by running downhill, which is characterized by decreased muscle strength, increased serum creatine kinase (CK) activity and an inflammatory response [6,7,8]. The use of dietary supplements is common among ultra-endurance runners to improve performance, prevent damage and accelerate post-race recovery [9]. Among them, supplements containing antioxidant compounds have been studied for years for their possible protective effects against oxidative damage induced in various types of races [10,11,12].

Antioxidants are compounds that help to protect cellular organs from oxidative damage caused by free radicals and other reactive oxygen species (ROS). The increase in these antioxidant molecules supports the hypothesis of a compensatory mechanism to avoid a situation of oxidative stress generated by an increase in ROS. While endogenous antioxidants are the first line of defense against free radicals, exogenous antioxidants provided in the diet act as a second line of defense and offer further protection [13]. Beetroot, which contains high levels of nitrates and phytochemicals including betalain, ascorbic acid, carotenoids, phenolic acids and flavonoids [14], is a powerful antioxidant that seems to improve exercise performance [15,16]. Less is known about its effects on the recovery after ultra-endurance sports. Therefore, the present study investigated the effect of beetroot supplements during the performance of endurance exercise on plasma oxidative stress, muscle damage and systemic inflammation at the end of the IX Penyagolosa Trails CSP 2019 (Penyagolosa Trails, n.d.) and more specifically on the recovery during the following two days.

## 2. Materials and Methods

### 2.1. Study Design

This research is an observational and longitudinal study. It was carried out by means of interviews and blood samples collected from 32 runners who finished the IX Penyagolosa Trails CSP^®®^ (Castelló-Penyagolosa) race and during the two following days. This race is an ultra-trail of 107.4 km with an elevation gain of 5600 m uphill and 4400 m downhill. The start is at 40 m above sea level and the finish is at 1280 m above sea level.

### 2.2. Participants

Thirty-two amateur ultra-endurance athletes (thirteen females and nineteen males) were recruited to participate in the study. All volunteers were fully informed of the procedure and informed consent was obtained from all subjects involved in the study. They were also allowed to withdraw from the study at any time.

### 2.3. Data Collection

A questionnaire was used to collect demographic information, training and competition history and consumption of beetroot supplements during the race. Muscular strength was measured using the squat jump (SJ) test, a validated research test based on three parameters (body mass, jump height and thrust distance) that allows accurate assessment of the strength, speed and power developed by the extensor muscles of the lower extremities during squat jumps [17]. Before testing, participants were briefed and instructed regarding how to proceed, which was performed before the race and within 15 min after the race.

Blood samples were obtained during race number collection (8 to 6 h before the commencement of the race), immediately after crossing the finish line and at 24 and 48 h post-race. Subsequently, the samples underwent centrifugation at 3500 rpm for ten minutes and were stored at 4 °C during transportation to Vithas Rey Don Jaime Hospital in Castellon.

### 2.4. Intervention

At the hospital, the samples were processed using the modular platform Roche/Hitachi clinical chemistry analyzer Cobas c311 (Roche Diagnostics, Penzberg, Germany), following previously published protocols [18,19]. Lactate dehydrogenase (LDH) and creatine kinase (CK) were analyzed to evaluate muscle damage. C-reactive protein (CRP) was measured as an indicator of acute inflammatory response [20]. The oxidative stress biomarkers used in the present investigation were GR, GPx, MDA and CG, which were analyzed in the laboratories of Universitat Jaume I as follows:

GPx activity, responsible for catalyzing the oxidation of glutathione (GSH) to its disulfide form (GSSG) in the presence of hydrogen peroxide (H_2_O_2_), was quantified spectrophotometrically following the methodology described by Lawrence et al. [21]. The assay was conducted towards hydrogen peroxide by monitoring the oxidation of nicotinamide adenine dinucleotide phosphate (NADPH) at 340 nm. The reaction mixture comprised 240 mU/mL of GSH disulfide reductase, 1 mM GSH, 0.15 mM NADPH in 0.1 M potassium phosphate buffer (pH 7.0), with 1 mM EDTA and 1 mM sodium azide. A 50 µL sample was introduced into the mixture and allowed to equilibrate at 37 °C for 3 min. The reaction was initiated by adding hydrogen peroxide, adjusting the final volume of the assay mixture to 1 mL.

GR catalyzes the reduction of GSSG to GSH, and its activity was determined spectrophotometrically using the method proposed by Smith [22]. Briefly, the sub-production of 2-nitrobenzoic acid obtained during the reduction reaction of GSSG was monitored at 412 nm. The GSSG reduction was started by adding 25 µL of brain sample to a solution containing DTNB 3 mM prepared in 10 mM phosphate buffer, 2 mM NADPH and 10 mM MEDTA in 0.2 M pH 7.5 phosphate buffer.

MDA concentration was determined using liquid chromatography, following a modification of the method outlined by Richard and colleagues [23], as previously documented [24]. In summary, 0.1 mL of the sample (or standard solutions prepared daily from 1,1,3,3-tetramethoxypropane) was mixed with 0.75 mL of the working solution (thiobarbituric acid 0.37% and perchloric acid 6.4%; 2:1, *v*/*v*) and heated to 95 °C for 1 h. After cooling for 10 min in an ice water bath, the flocculent precipitate was eliminated by centrifugation at 3200× *g* for 10 min. The supernatant was neutralized, filtered (0.22 µm) and then injected onto an ODS 5 m column (250 × 4.6 mm). The mobile phase consisted of 50 mM phosphate buffer (pH 6.0): methanol (58:42, *v*/*v*). Isocratic separation was achieved with a flow rate of 1.0 mL/min, and detection was performed at 532 nm.

Carbonyl groups (CG) were quantified to assess oxidative damage to proteins in blood samples. The CGs released during incubation with 2,4-dinitrophenylhydrazine were measured following the method outlined by Levine et al. [25], with certain modifications introduced by Tiana et al. [26]. In summary, the samples underwent centrifugation at 13,000× *g* for 10 min. Subsequently, 20 mL of brain homogenate was placed in a 1.5 mL Eppendorf tube, and 400 mL of 10 mM 2,4-dinitrophenylhydrazine/2.5 M hydrochloric acid (HCl) and 400 mL of 2.5 M HCl were added. This mixture was incubated for 1 h at room temperature. Protein precipitation was achieved using 1 mL of 100% TCA, washed twice with ethanol/ethyl acetate (1/1, *v*/*v*) and centrifuged at 12,600× *g* for 3 min. Finally, 1.5 mL of 6 N guanidine, pH 2.3, was added, and the samples were incubated in a 37 °C water bath for 30 min and then centrifuged at 12,600× *g* for 3 min. The carbonyl content was calculated from peak absorption (373 nm) using an absorption coefficient of 22,000 M^−1^ cm^−1^ and expressed as nmol/mg protein.

### 2.5. Data Analysis

Biochemical results obtained immediately post-race were adjusted by employing the Dill and Costill method [27], using hematocrit and hemoglobin to determine the magnitude of plasma volume changes after the race in each participant.

Beetroot antioxidant supplementation or not was considered an independent/secondary variable. Dependent variables corresponding to GR (IU/mL), GPx (μmol/L × min), CG (nmol/mL), MDA (μM), LDH (IU/l), CK (IU/l) and CRP (mg/dl) were obtained by blood analysis before the race, at the finish line and at 24 and 48 h later. Furthermore, the values of all damage parameters for each participant were related to the individual baseline values to define the delta values (Δ) according to the following equation:

Δ (fold increase) = (post-race value − Pre-race value)/Pre-race value

### 2.6. Statistical

Statistical analyses were carried out using SPSS software (IBM SPSS Statistics for Windows, Version 28.0 IBM Corp, Armonk, NY, USA). Results were presented as mean values ± SE. The normal distribution of the variables was verified by the Shapiro–Wilk test (*p* > 0.05). The possible effects of beetroot on indicators of oxidative stress, muscle damage and inflammatory response at the end of the race and after 24 and 48 h, as well as loss of muscular strength (SJ) at the finish line, were analyzed by means of two-way ANOVA. The analysis of variance of the obtained data was performed by the Levene test using the LSD test as a post hoc test when the data showed homogeneity in their variances (*p* < 0.05) or a Dunnet T3 test when variances differed. Statistical significance differences were set at the *p* < 0.05 level. The effect size was estimated by calculating Cohen’s d.

## 3. Results

### 3.1. Runners Characteristics

A descriptive analysis of the participant characteristics is presented in Table 1. Thirty-two of the participants in this study reached the finish line, of which thirteen were women and nineteen were men. The fastest time was 14 h 15 min, the slowest was 27 h and the average time was 21,38 h. The average age of the participants was approximately 41 years, with a minimum age of 31 and a maximum age of 53. The subjects were amateur runners, and there were no significant differences in training characteristics between women (13) and men (19). On the other hand, the men had significantly higher pre-race values for weight, BMI and muscle mass compared to the women. Regarding beetroot antioxidant supplementation, as shown in Table 1, a total of six participants consumed beetroot during the race, three of whom were women and the rest men.

### 3.2. Effect of Beetroot Intake on the Runners’ Oxidative Status

**Figure 1:**
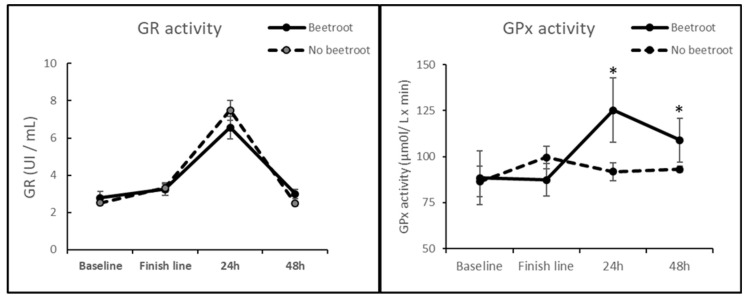
Effect of beetroot supplementation on GR and GPx activity. * *p* < 0.05 vs. no beetroot supplementation group.

Regarding the serum oxidative status of the runners, as shown in Table 2 and Figure 1, the GPx activity was significantly higher in the beetroot group 24 and 48 h after the end of the race when compared to its activity in runners who did not consume this supplement. In addition, the time course study of GPx activity shows that it is increased 24 h after the race compared to the finish line values in the beetroot group, but no changes over time were observed in the runners who did not consume beetroot. The GR activity also was significantly increased 24 h after the race in both groups, but beetroot supplementation did not affect the activity of this antioxidant enzyme.

Concerning oxidative damage to lipids (MDA) and proteins (CG) (Table 3), it is remarkable that the lipid oxidative damage (MDA levels) increased at the finish line and 48 h post-race in runners who did not consume beetroot, whereas supplementation with this dietary supplement protected against lipid peroxidation. Similar results were obtained for protein oxidative damage because, although the CG content increased in both groups at the finish line, faster recovery is observed in the beetroot group since the 24 h GC values are no longer statistically different from the pre-race values (Table 3).

### 3.3. Effect of Beetroot Intake on Runners’ Muscle Damage and Inflammatory Processes

Table 4 summarizes the effects of beetroot consumption on the muscle damage parameters (CK and LDH values) in the runners participating in the IX Penyagolosa Trails CSP.

**Figure 2:**
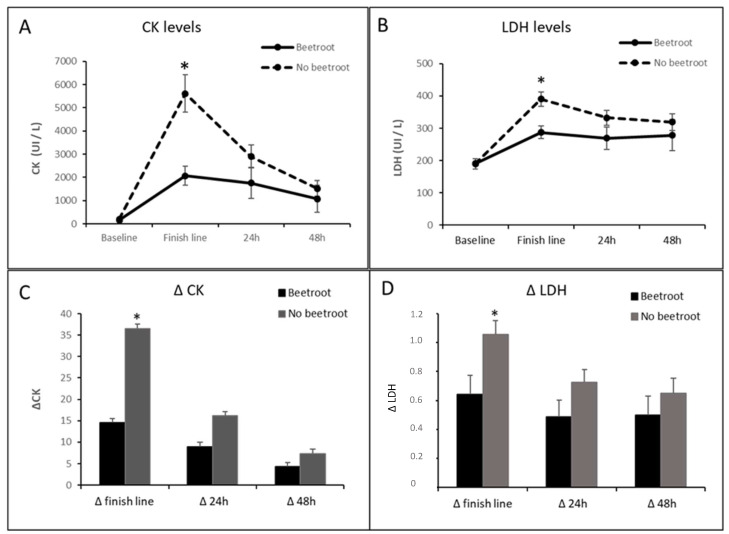
Effect of beetroot supplementation on CK and LDH. * *p* < 0.05 vs. beetroot supplementation group. (**A**). Blood Ck levels; (**B**). Blodd LDH levels; (**C**). Blood CK Delta values; (**D**). Blodd LDH Delta values.

When the effect of beetroot intake on muscle damage was studied, it was observed that both the CK and LDH levels were significantly higher at the finish line in the runners who did not consume beetroot compared to those who did (Figure 2A,B). Similar results were obtained with respect to the CK and LDH delta values (Figure 2C,D).

Beetroot intake did not affect CRP levels, suggesting that this supplement does not modulate inflammatory processes induced during this type of ultra-trail (Table 4).

**Figure 3:**
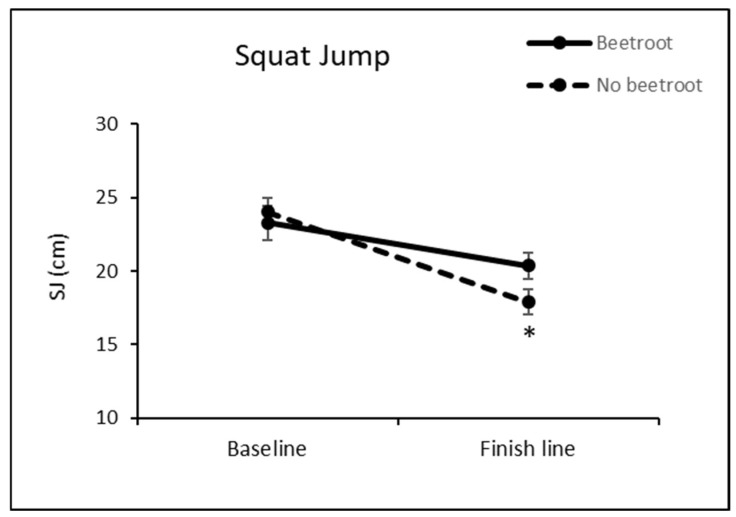
Effect of beetroot supplementation on SJ performance. * *p* < 0.05 vs. baseline value.

Finally, with regard to the loss of muscular strength (SJ test), the two-way ANOVA shows a significant loss of muscular strength in the group that did not consume beetroot at the end of the race. This was not observed in the group that reported beetroot intake during the race (Figure 3).

## 4. Discussion

The role of beetroot supplementation as a modulator of exercise performance and its potential beneficial effect on recovery from muscle damage induced by high-intensity exercise have been the subject of numerous studies. In fact, the International Olympic Committee consensus statement on dietary supplements and high-performance athletes [28] lists a number of supplements to improve muscle recovery and muscle damage, including beetroot. However, the results of these studies are controversial, with some studies showing a potential beneficial effect and others finding no significant effect [29,30,31,32]. The present work aimed to ascertain if beetroot consumption has a role on the recovery of oxidative status and muscle damage in ultra-endurance runners. For this purpose, we carried out an observational and longitudinal study in which six runners reported beetroot intake during the IX Penyagolosa Trails CSP^®®^ (Castelló-Penyagolosa) race (107.4 Km). The primary limitation of this study stems from the low participation rate, with only six subjects consuming beetroot, posing challenges for statistical analysis. However, this observation highlights the widespread adoption of beetroot consumption among athletes, thereby advocating for further investigations with a larger sample size to elucidate the efficacy of this supplementation.

Concerning the oxidative status of the runners, a time-course analysis of GPx and GR activity and oxidative damage to lipids and proteins (MDA and CG, respectively) was performed at the finish line, 24 h and 48 h after the race. As seen in Figure 1, the activity of the antioxidant enzyme GR was significantly increased 24 h after finishing the race in all the participants; however, the GPx activity only increased in those runners who reported beetroot consumption during the race (Figure 1). Interestingly, we have previously reported that GPx activity is not modified after this ultra-trail race [8], so it seems that beetroot consumption could specifically enhance the activity of this enzyme.

Furthermore, we also report an increase in oxidative damage to lipids and proteins (MDA levels and CG content) at the end of the race in the runners who did not consume beetroot, which is not observed in those who did, again suggesting that beetroot consumption could protect against oxidative damage, probably due to its antioxidant properties [33]. There is no consensus in the scientific literature regarding the effects of beet on oxidative lipid damage associated with exercise. There appear to be differences depending on the type of exercise, its duration and intensity as well as the timing of beet consumption and the duration of supplementation. Thus, in agreement with our results, Hasibuan and collaborators reported that the administration of beet juice to athletes during their maximal physical activity reduced MDA levels [34]. In contrast, Kozłowska et al. showed that long-term beetroot juice supplementation increased lipid peroxidation in elite fencers [35]. Clifford et al. also failed to find a positive effect of beetroot juice on the recovery of oxidative damage to proteins between two repeated sprint tests when beetroot juice was supplemented after the sprint tests [29].

We also analyzed the effect of beetroot intake on runners’ muscle damage and inflammatory processes. As shown in Table 3 and Figure 2, beet consumption protects against muscle damage by significantly attenuating the increase in CK and LDH. Other authors have described similar results in a variety of sports modalities, such as fencers [35], but, in others, such as soccer players [30] or even marathon runners [36], beetroot failed to achieve this effect. It is remarkable, however, that those marathon runners were only supplemented with beetroot after the race. Therefore, our findings suggest that the efficacy of beet supplementation for mitigating muscle damage may be more pronounced when administered during the race rather than in the post-race period. Conversely, our examination did not reveal a statistically significant impact of beet consumption on inflammation markers (Table 4). In this context, although beetroot has been broadly described as an anti-inflammatory agent [37], the prevailing body of published research concerning its anti-inflammatory impact over the consequences of physical activity is aligned with our findings [30,33,36].

Finally, beetroot intake mitigated the significant loss of muscular strength (SJ test) in the group that did not consume beet at the end of the race (Figure 3). Similar to the observed glutathione peroxidase (GPx) activity, our prior investigations demonstrated a lack of statistically significant decline in muscle strength subsequent to the Penygolosa Trail CSP [8]. Nevertheless, upon segmenting the sample to assess the impact of beet consumption, a significant reduction in muscle strength was evident within the non-beet-consuming group, highlighting the potential of beet supplementation to mitigate such strength losses. The ergogenic potential of beetroot has been addressed in previous literature [38,39]. However, contrary to our findings, specific studies have documented instances where beetroot supplementation did not fully recover the exercise-induced decline in muscular strength among female volleyball players [32] or following a marathon [36].

Practical application: Supplementation is prevalent across all tiers of sports; however, improper use of certain supplements may harm the athlete’s health and their performance. Therefore, prior to supplement recommendation, a comprehensive nutritional evaluation should be conducted. Products claiming to boost performance represent the most abundant category marketed to athletes, yet there remains limited understanding regarding their effectiveness in post-competition recovery. Hence, research such as the present study, especially in amateur competitions that are less regulated, is essential to improve our understanding of various uses of supplements such as beetroot.

## 5. Conclusions

Although there is extensive scientific evidence that short-term beetroot supplementation can accelerate recovery from muscle damage after physical activity, additional investigation is required to elucidate whether an extended duration of supplementation (spanning a few days prior to and following exercise) could also facilitate the recuperation of indicators related to muscular damage, inflammatory response and oxidative stress [31]. Interestingly, beetroot is always mentioned among the supplements recommended for ultra-endurance athletes [40], and, indeed, six of the thirty-two participants in our study reported beetroot intake during the race. However, there is a lack of studies showing its effects on restoring muscle damage and oxidative damage markers in ultra-endurance runners. Ultramarathons have become increasingly popular in recent years. These extremely long races defy our physiological systems and expose the athlete to an extremely high degree of functional and structural damage. The results of this observational study suggest that beetroot consumption during an ultramarathon may protect against oxidative stress and muscle damage, thereby attenuating the loss of muscle strength. Given the favorable outcomes observed, it is imperative to conduct an intervention study to substantiate the effect of beetroot supplementation under controlled conditions of dosage and duration.

## Figures and Tables

**Table 1:** Runner characteristics.

	Total (n = 32)	Women (n = 13)	Men (n = 19)
Average time (h)	21.38 ± 0.61	21.39 ± 0.88	21.36 ± 0.89
Average age (years)	40.9 ± 1.0	42.2 ± 1.7	40.1 ± 1.2
Years runninng	8.0 ± 0.5	8.0 ± 0.6	8.1 ± 0.9
Training hours/week	9.6 ± 4.6	10 ± 0.9	9 ± 1.3
Pre-race weight (kg)	66.1 ± 1.9	55.7 ± 1.4	73.2 ± 1.5 *
BMI	22.9 ± 0.4	21.7 ± 0.6	23.8 ± 0.4 *
Pre-race % Muscular Mass	84.1 ± 1.1	79.1 ± 1.3	87.6 ± 0.8 *
Beetroot consumption (nº of runners)	6	3	3

* *p* < 0.05 vs. women.

**Table 2:** Antioxidant enzyme activity.

	With Beetroot (n = 6)	Without Beetroot (n = 26)
GPx baseline (µmol/L × min)	88.4 ± 14.6	86.5 ± 8.3
GPx finish line (µmol/L × min)	87.4 ± 8.8	99.5 ± 6.2
GPx 24 h post-race (µmol/L × min)	125.3 ± 17.4 ^†^ (0.70)	91.7 ± 4.9 * (1.13)
GPx 48 h post-race (µmol/L × min)	109.0 ± 11.8	93.1 ± 1.5 * (1.15)
GR baseline (UI/mL)	2.7 ± 0.3	2.5 ± 0.1
GR finish line (UI/mL)	3.2 ± 0.3	3.3 ± 0.2
GR 24 h post-race (UI/mL)	6.5 ± 0.6 ** (2.09)	7.4 ± 0.5 ** (2.64)
GR 48 h post race (UI/mL)	2.9 ± 0.2	2.4 ± 0.1

* *p* < 0.05 vs. beetroot intake; ^†^ *p* < 0.05 vs. finish line values; ** *p* < 0.05 vs. baseline, finish line and 48 h post-race. In parentheses: effect size calculated using Cohen’s d.

**Table 3:** Oxidative damage to lipids (MDA) and proteins (CG).

	With Beetroot (n = 6)	Without Beetroot (n = 26)
MDA baseline (µM)	1.1 ± 0.5	0.6 ± 0.1
MDA finish line (µM)	1.0 ± 0.2	1.3 ± 0.1 ^†^ (0.80)
MDA 24 h post-race (µM)	0.8 ± 0.1	0.8 ± 0.1
MDA 48 h post-race (µM)	1.2 ± 0.1	1.4 ± 0.1 ^†^ (0.94)
CG baseline (nmol/mL)	1.26 ± 0.09	1.4 ± 0.1
CG finish line (nmol/mL)	2.7 ± 1.1	2.1 ± 0.2 ^†^ (0.54)
CG 24 h post-race (nmol/mL)	1.7 ± 0.3	1.9 ± 0.12
CG 48 h post-race (nmol/mL)	1.6 ± 0.4	2.2 ± 0.3 ^†^ (0.43)

^†^ *p* < 0.05 vs. baseline values. In parentheses: effect size calculated using Cohen’s d.

**Table 4:** Effect of beetroot supplementation on the muscle damage and inflammation variables.

	With Beetroot (n = 6)	Without Beetroot (n = 26)
CK baseline value (UI/L	159.5 ± 33.5	213.9 ± 47.4
CK finish line value (UI/L)	2071.7 ± 406.7	5616.01 ± 816.1 *^†^ (0.98/1.30)
CK 24 h post-race value (UI/L)	1758.5 ± 663.7	2901.6 ± 503.4 ^†^ (1.05)
CK 48 h post race value (UI/L)	1076.2 ± 588.5	1521.6 ± 338.3 ^†^ (0.77)
LDH baseline value (UI/L)	185.2 ± 15.2	190.4 ± 5.9
LDH finish line value (UI/L)	299.4 ± 20.7 ^†^ (1.72)	390.6 ± 22.4 *^†^ (0.85/2.0)
LDH 24 h post-race value (UI/L)	277.0 ± 32.4	332.5 ± 23.2 ^†^ (1.41)
LDH 48 h post-race value (UI/L)	282.83 ± 43.28	318.92 ± 25.61 ^†^ (1.12)
CRP baseline value (UI/L)	0.11 ± 0.05	0.18 ± 0.09
CRP finish line value (UI/L)	1.87 ± 0.47	2.03 ± 0.25
CRP 24 h post-race value (UI/L)	3.60 ± 0.63	3.63 ± 0.49
CRP 48 h post-race value (UI/L)	2.17 ± 0.45	1.85 ± 0.25

* *p* < 0.05 vs. beetroot intake; ^†^ *p* < 0.05 vs. baseline values. In parentheses: effect size calculated using Cohen’s d.

## Data Availability

The raw data supporting the conclusions of this article will be made available by the authors on request.
